# Metals in Fishes from Yongshu Island, Southern South China Sea: Human Health Risk Assessment

**DOI:** 10.1155/2017/2458293

**Published:** 2017-10-19

**Authors:** Zhai Wu, Ye Xu, Minggang Cai, Sha-Yen Cheng, Huorong Chen, Dongren Huang, Kai Chen, Yan Lin, Tianyao Li, Mengyang Liu, Hengxiang Deng, Minjie Ni, Hongwei Ke

**Affiliations:** ^1^Fujian Provincial Key Laboratory for Coastal Ecology and Environmental Studies, Xiamen University, Xiamen 361102, China; ^2^Coastal and Ocean Management Institute, Xiamen University, Xiamen 361102, China; ^3^College of Ocean and Earth Science, Xiamen University, Xiamen 361102, China; ^4^State Key Laboratory of Marine Environmental Science, Xiamen University, Xiamen 361102, China; ^5^College of Ocean Science and Resource, National Taiwan Ocean University, Keelung 20224, Taiwan; ^6^The Monitoring Center of Marine Environment and Fishery Resources, Fujian Provincial Department of Ocean and Fisheries, Fuzhou 350003, China

## Abstract

In order to assess the bioaccumulation of metals associated with gender, tissues, and their potential ecological risk, four species of fish were collected from the Yongshu Island in the Southern South China Sea. Metals and stable Pb isotopes in their tissues (muscle, gill, liver, intestine, and ovary) were determined. The concentrations of metals (mg/kg, dry weight) in these species were ND–21.60 (Cd), 1.21–4.87 (Cr), 0.42–22.4 (Cu), 1.01–51.8 (Mn), 0.30–3.28 (Ni), 6.04–1.29 × 10^3^ (Zn), 14.89–1.40 × 10^3^ (Fe), and 0.22–3.36 (Pb). In general, the liver and intestine absorbed more metals than the other tissues. Metals accumulation can be influenced by gender and feeding behavior and in fact, female fish and dietary exposure are more prone to accumulate metals. In addition, Pb isotopic ratios indicated that all species had significant biological fractionation, which may not make them good tracers for source identification. The metal concentrations of most samples were lower than the national standard values of the FAO (USA), which suggested that human consumption of these species may not cause health risks. However, since the surrounding areas are developing rapidly, the potential environmental risk of metals will intensify and should receive more attention.

## 1. Introduction

Metals, a class of contaminants in aquatic ecosystems, can cause harm to marine organisms and to humans consuming polluted organisms [1–4]. Metals in the marine environments have both natural and anthropogenic sources. The latter, such as smelting, sewage, disposal of fuel burning, and recreational activities, contribute to increasing risks to the aquatic environment due to the rapid development of urban and industrial activities [5]. Via river discharge or atmospheric deposition, which is the most important pathway for the marginal sea, metals enter into aquatic environments and are continually concentrated in food chain through bioaccumulation and biomagnification [1, 6–8].

Heavy metals in excessive concentrations present some toxicity to the ecosystem owing to their persistent and nonbiodegradable properties. Exposure to heavy metal contaminants causes chronic DNA damage, degeneration of tissues and the nervous system, and interference with ion homeostasis [7, 9]. For example, Pb exposure results in anemia and an impact on hemoglobin synthesis [8, 10]. Cd exposure is related to nephropathy, lung cancer, osteoporosis, abnormal hemopoiesis, and various others [11–13]. The essential metals in excess levels are also toxic to organisms [14, 15]; for example, a high level of Cu influences erythrocyte function [16].

Marine organisms, especially fish at the top of the food chain, can assimilate large amounts of contaminants and store them in their tissues [1, 5]. Thus, fish are widely used as bioindicators of the aquatic environment, indicating environmental contamination [1, 3, 17, 18]. The processes of metals accumulation in fish tissues depend on the species, gender, size, metabolic activity of the fish, and the method of metal exposure (e.g., dietary and water exposure) [1, 8, 19, 20]. The accumulation of metals in tissues shows enormous variability owing to specific tissue functions, especially metallothioneins (MTs) [21]. MTs, regarded as a biomarker and a cysteine bonding metal, are important for the homeostatic and detoxification of metals [22, 23]. Furthermore, fishes containing high quality protein have great popularity and are consumed in large quantities owing to their benefit to human health [24]. However, fishes contaminated by metals are potentially toxic for humans [25]. In particular, fish can accumulate metals throughout the food chain and generate the toxic effect on human health. Therefore, research into the accumulation of metals in fish tissues is a necessary and good tool to monitor pollution in the environment and to assess the safe threshold for human consumption.

Pb isotopic ratio is an efficient tool to trace sources and pathways of Pb. The stable isotopes of Pb are composed of ^204^Pb, ^206^Pb, ^207^Pb, and ^208^Pb, of which the latter three are products of radiogenic elements. The composition of Pb isotopes depends on the decay rate and the amount of parent isotopes, and its fractionation does not present in physical and chemical processes [26]. The different sources of Pb pollution and ore deposits have unique Pb isotopic ratios, and therefore, many previous studies used isotopic fingerprinting to identify the source of Pb pollution in aerosols [27, 28], sediments [29–31], and organisms [32–34]. However, the study about Pb isotopic ratios in different fish tissues is scarce so far, because of biological fractionation [32]. Thus, our study set out to identify how much trust can be placed on Pb isotopic fingerprinting in the source apportionment of fish.

The Nansha Islands are one of the major archipelagos in the South China Sea (SCS), which consist of some islands, islets, cays, and a large number of coral reefs. This archipelago possesses rich natural resources, for example, fisheries, guano, natural gas, and oil reserves, which have the potential to contribute significant economic value to the neighboring countries [35]. Yongshu Island is located on the western edge of the Nansha Islands and is the third largest artificial island (3.06 km^2^) in this area. Considering the circumstance of the big increase of fishing and culturing activities in the SCS and fast socioeconomic developing of surrounding regions, such intensive human activities on Yongshu Island will discharge pollutants into the local environment and threaten marine organisms [36, 37]. Furthermore, the fishes captured/cultured there will be consumed by the people living in the neighboring countries, and this raises concern about the potential health risks [38, 39]. As far as we can ascertain, research concerning the concentration and source of metals in fish or other organisms from the waters near Yongshu Island is scarce.

Based on the measured metal concentrations in different tissues (including muscle, gill, liver, intestine, and ovary) of four species fish living there, the objectives of this study included the following: (1) to discuss the metal concentrations in fish tissues; (2) to identify the influence of gender and uptake way on the accumulation of metals in fish; (3) to clarify the influence of Pb isotope fractionation in different tissues; and (4) to assess the human consumption risk by comparing our results with international guidelines for metals.

## 2. Materials and Methods

### 2.1. Samples Collection

**Figure 1:**
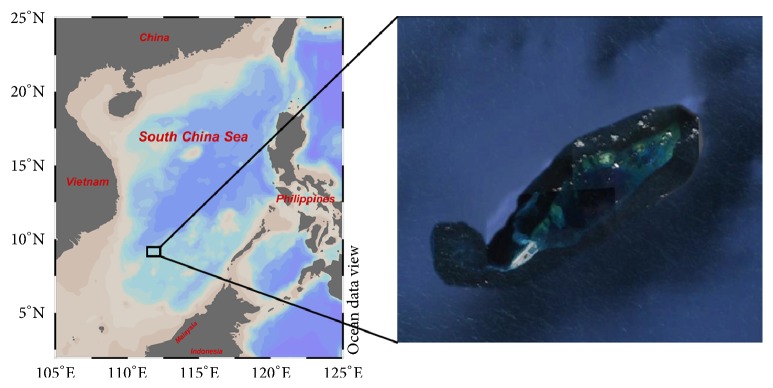
The location map of Yongshu Island (9°32′–9°42′N, 112°52′–113°04′E), South China Sea. The sampling sites of fishes were distributed outside the island.

Yongshu Island (Figure 1), located in the SCS (9°32′–9°42′N, 112°52′–113°04′E), is a typical semiclosed atoll with an area of about 110 km^2^ and a depth of 200 m. The reef is in the shape of a spindle, whose length extends in an NEE-SWW direction for about 25 km, and the width extends in an NW-SE direction for about 6 km.

This research was performed on board of the R/V* “SHIYAN III”* from the South China Sea Institute of Oceanology in August 2013. Twenty-four fish individuals from four species (including* Gnathodentex aureolineatus (G. aur)*,* Oxycheilinus diagrammus (O. dia)*,* Melichthys vidua (M. vid)*, and* Lutjanus kasmira (L. kas)*) were collected by the fishing-line near the island.

### 2.2. Laboratory Treatment and Instrumental Analysis

The collected samples were washed with deionized water, and their body weights and lengths were recorded. Then the samples were stored in polyethylene bags and kept in a freezer (−20°C) prior to analysis. Each fish was dissected using a cleaned stainless-steel knife to obtain gill, liver, muscle, heart, and ovary tissues. The* G. aur* were distinguished for gender by recognition of their genitals. Each sample was freeze-dried and then powdered in an agate mortar.

The metal concentrations in samples of each fish were analyzed based on the USEPA (1990) method with some modifications [46, 47]. Briefly, the powdered samples (0.200 ± 0.001 g, dry weight) were digested using 2.0 mL of HNO_3_ in Teflon, and the solution was heated at 60°C until the froth disappeared. Subsequently, the residues were successively digested with 0.5 mL H_2_O_2_, 1.0 mL H_2_O_2_, and 1.0 mL HNO_3_ with 1.5 mL H_2_O_2_ at 170°C. After digestion, the solution was diluted to 25 mL with ultra-pure water. After overnight settling, the solution was transferred into a polyethylene tube and stored at 4°C. At the same time, to estimate the interference, accuracy, and precision of instructional analysis, the blanks and standard reference material (cod, DORM-4) from the National Research Council of Canada were processed along with the samples.

The concentrations of Zn and Fe were measured using flame atomic absorption spectrometry (FAAS, Thermo Electron M6), while concentrations of Cu, Pb, Cr, Cd, Mn, and Ni were determined using inductively coupled plasma-mass spectrometry (ICP-MS, Agilent 7500cx). The samples of fish tissues were also analyzed for their Pb isotopic composition using ICP-MS. All analytical solutions for Pb isotope analysis were diluted to about 25 *μ*g/L Pb with 1% HNO_3_ and were determined using ICP-MS.

### 2.3. QA/QC

The average recoveries of most metals were around 92–114%, while the recovery of Pb and Cr was 108 and 109% (see Table 1). The quality control standards were used every 10 samples to ensure no contamination and drift from FAAS and ICP-MS analysis.

For Pb isotopic analysis, a national standard reference material (GBW04425, China) was analyzed for calibration and analytical control. Each sample was determined five times, and the relative standard deviation of each sample was <0.5%. The average measured ratios of ^204/206^Pb, ^207/206^Pb, and ^208/206^Pb were 0.1152 ± 0.000002, 0.4692 ± 0.000041, and 1.0063 ± 0.00012, and the standard values were 0.1156, 0.4694, and 1.0065.

### 2.4. Statistical Analysis

Statistical analysis was performed using SPSS 22.0 (International Business Machines Corp.). One-way ANOVA was applied to determine the variation of metals in different fish species and tissues. The relationships among metals were studied using Pearson correlation analysis. In addition, principal component analysis was used to reduce the dimensionality of the data and to further identify the relationships of metals in different fish species.

### 2.5. Calculation

The provisional maximum tolerable daily intake (PMTDI) is proposed by the WHO/Joint Expert Committee on Food Additives (JECFA), which represents the maximum value of intake in food by humans. According to the report by WHO/JECFA, the PMTDI of Cu, Fe, Mn, Zn, Cd, Ni, and Pb are 50 *μ*g/(kg BW)/day, 4800 *μ*g/(kg BW)/day, 8 *μ*g/(kg BW)/day, 100 *μ*g/(kg BW)/day, 0.5 *μ*g/(kg BW)/day, 5.0 *μ*g/(kg BW)/day, and 3.57 *μ*g/(kg BW)/day [48–50]. The maximum value for human consumption is calculated as follows [17]:

(1) $$\begin{array}{c} {MSC}_{X}=\frac{\left(BW*{PMTDI}_{X}\right)}{C_{X}}, \end{array}$$

where *C*_*X*_ is the concentration of element *X* (*μ*g/g), MSC_*X*_ is the maximum safe consumption by humans (g fish/day), and BW is body weight (60 kg).

## 3. Results and Discussion

### 3.1. Variation of Metal Concentrations among the Fish Species

**Figure 2:**
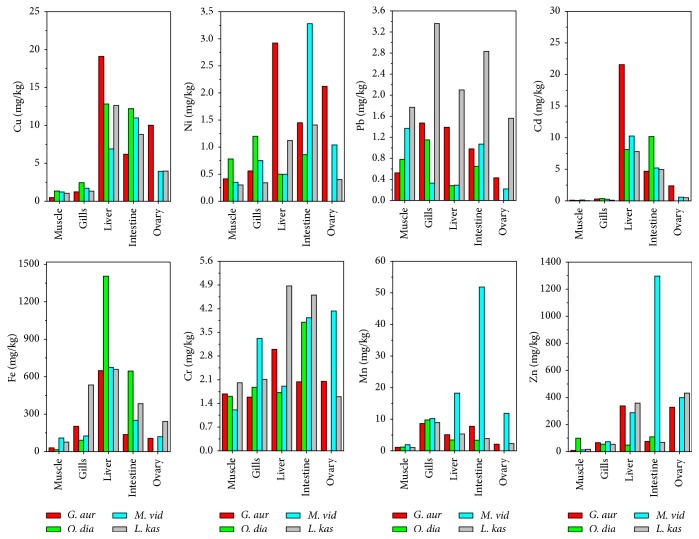
The distribution of metals in muscle, gill, liver, intestine, and ovary from four fish species.

Concentrations of eight metals in* G. aur*,* O. dia*,* M. vid*, and* L. kas* (whose total length varied from 17.38 cm to 22.00 cm) were analyzed. As shown in Tables 2 and 3, the concentrations of Cd, Cr, Cu, Mn, Ni, Zn, Fe, and Pb ranged from ND–21.60, 1.21–4.87, 0.42–22.4, 1.01–51.8, 0.30–3.28, 6.04–1296.26, 14.89–1405.31, and 0.22–3.36 mg/kg. According to the results of one-way ANOVA, there were significant interspecies variations in Pb accumulation (*p* < 0.01) (Figure 2). Fe and Zn had the highest concentrations in different fish species, which was probably because they were essential elements for the organism. For example, Zn is important for gene proteins (e.g., zinc finger protein), and Fe is an indispensable element for heme enzymes and ribonucleotide reductase [19, 51, 52]. Mn, Cu, Cr, and Ni are also essential for organisms but showed lower levels probably due to the unavailable high molecular mass complexes and the formation of a less liposolubility organic complex [53]. Pb and Cd are nonessential elements for organisms, but the value of Pb in* L. kas* was generally higher than in the other species, reflecting their diet, growth stage, and the surrounding environment [47].

In general, the concentrations of metals in aquatic organisms significantly varied with different areas and fish species (Table 4). For example, the concentrations of metals in this study area were generally higher than those in the western SCS [39]. Compared with metals concentrations in the Pearl River Estuary, the concentrations of Mn, Cr, Cu, Ni, and Pb were higher in the Yongshu Island [40]. The concentrations of Mn, Cr, Cu, and Ni in fishes in the south of Brazil were higher than in other regions [41], while the concentrations of Fe and Zn in the Yongshu Island were the highest. Concentrations of Cu, Ni, and Pb in fishes were lower than in aerosol and sediments in the SCS, while concentration of Zn in fishes was higher [5, 44]. The concentrations of Cd and Cr in fish were lower than in sediments [44]. These results from other studies indicated that different environments, fish species, and water quality can influence the concentration of metals in fish [54].

### 3.2. Different Bioaccumulation in a Specific Gender

There was a significant difference of metal concentrations between different genders in* G. aur* (Table 2), although the male organ was too small to dissect. Generally, the mean concentrations of most metals were higher in female* G. aur*, which were also found in* Lethrinus lentjan* from the Arabian Gulf and* Fundulus heteroclitus* from New Jersey and Long Island, USA [1, 55].

As for the specific tissues, there were no significant differences between males and females. On the one hand, most metals presented higher concentrations in female muscle and gill; only Ni in muscle and Fe and Cr in the gill were higher in male. This contrasted with the metal distribution in the intestine, where the male accumulated more metals except Fe, Zn, and Pb. Concentrations of Fe, Zn, and Cu in livers were higher in female, whereas Mn, Pb, and Cr were higher in male. On the other hand, certain metals in some tissues showed no difference regarding gender, such as Ni in the gill and liver, Cd in the liver, and Mn in the muscle. The accumulation of metals is influenced mostly by hormonal activities, and besides that, growth rate, diet, and environment also need to be considered [1, 56]. The female and male fish had different concentrations of steroid hormone, and the highest concentrations were found in the liver. Metals can be combined with steroid hormone and then stored in ovary or sperm to result in the different metal concentrations in each gender [18].

### 3.3. Accumulation of Metals in Specific Tissues

The maximum concentrations of Zn, Mn, and Ni were shown in the intestine of* M. vid* (Figure 2), while those of Fe, Cd, and Cr were found, respectively, in the liver of* O. dia*, female* G. aur*, and* L. kas*. Fe and Cd had significantly higher concentrations in the liver than in other organs (*p* < 0.01), except in the intestine of* O. dia* (*p* < 0.05). The concentration of Cu varied significantly with different organs (*p* < 0.05), and a higher concentration was observed in the liver than in the muscle, gill, or ovary. The liver is an important organ for accumulation, metabolism, and detoxification with a large number of MTs, which are regarded as biomarkers and as cysteine bonding metals [22]. The observed high concentrations of metals in the liver were related to the formation of MTs and complexing with enzymes. Therefore, liver of fishes is normally recognized as an indicator of environmental pollution and is used to determine the effect of pollution.

In addition to the liver, the intestine can indicate environmental stress from metals. Figure 2 shows that the intestine was the main organ to accumulate metals, especially in* O. dia* for Fe (645.35 mg/kg) and Cd (10.19 mg/kg);* M. vid* for Mn (51.83 mg/kg), Ni (3.28 mg/kg), and Zn (1296.26 mg/kg); and* L. kas* for Cr (4.60 mg/kg) and Pb (2.83 mg/kg). The intestine is a site for dietary exposure with abundant MTs and so dietary metals can be captured by the MTs and accumulated in the intestinal epithelia [20].

Similar to the intestine, the gill is a major organ for metal uptake during water exposure and is the first direct contact with sea water to take in pollutants [57]. Compared with the liver and intestine, the accumulation of metals in the gill was lower. The higher concentrations were present in* O. dia* for Cu (2.45 mg/kg);* L. kas* for Fe (533.80 mg/kg) and Pb (3.36 mg/kg);* G. aur* for Zn (92.62 mg/kg); and* M. vid* for Mn (10.21 mg/kg) and Cd (0.25 mg/kg). Mn showed a particularly high concentration due its bioavailability to fish [1]. In addition, the gill has the thinnest epithelium so facilitating ion exchange with other tissues. The* L. kas* accumulated more Pb in gill than other species, probably because the stress from pollutants influences enzymatic activities and ion exchange. In addition, the high concentration of Pb will cause the decreasing of enzymatic activity in fishes, and then it makes gill more exposed to contaminant [58].

Some metals, especially Zn and Cr, were found to accumulate in the ovary and may play an important role in the normal endocrine system of the organism [59]. However, a high concentration of Zn may result in abnormalities of the gonads in fish [56].

The concentrations of metals stored in muscle were the lowest. For example, concentrations of Cd in* O. dia* and* L. kas* were lower than the detection limit, but Mn and Ni were accumulated significantly less in muscle than in the intestine (*p* < 0.05). The reason may be that muscle is not an active tissue to take in nutritive materials compared with liver and gill [60]. However, unlike other metals, Pb concentration in muscle was not significantly lower than in other tissues, which may be attributed to the environment, species, metabolism activities, and eating habit.

### 3.4. Comparison between Dietary and Water Exposure

Generally, fish have two primary means of metal exposure: dietary exposure by the intestine and water exposure by the gill [20, 61]. As the osmoregulatory tissues of fish, the gill and intestine make first contact with the environment [62].

Most metals (Cd, Cr, Cu, and Zn) presented the highest concentrations in the intestine of all fish species (Tables 2 and 3), especially for the intestine in* M. vid*. On the other hand, Pb and Mn showed higher concentrations in the gill of the other three species, and high concentrations of Ni in* O. dia* and Fe in* L. kas* were also present in the gill. By way of conclusion, diet is the primary source of metal contamination in fishes, as noted in* Mullus barbarous* and* Salmo trutta* [20, 63]. The intestine has a large number of natural resistance associated macrophage proteins, and one of them is DMT1, which is related to the transportation of metals and could contribute to the interactions of Cu and Zn in the intestine [64]. On the other hand, metals may compete against each other to bond with DMT1; for example, Ni and Pb inhibit the absorption of intestinal Fe, and Cd competes with Fe^2+^ for uptake in the fish intestine [65].

### 3.5. Relationship between Metals in Fish Species

Significant positive relationships were found among Zn, Mn, and Ni (*p* < 0.01) as well as Cd and Cu (*p* < 0.01) in different tissues (Table 5). The concentrations of Cr and Zn also showed a significant positive relationship (*p* < 0.05).

The data concerning metals in different fish species were extracted to three principal components (PCs), and these components accounted for 72.785% of the total variance (Table 6). PC1 made up 35.323% of the total variance, which was composed of Mn, Zn, and Ni, representing bioavailability and essential elements for the marine organism. Cu and Cd had the highest loading on PC2 and explained 23.633% of the total variance. These two metals can both form stable chelates (e.g., tetrahedral MT) with some protein molecules and further affect the metabolic process of the organism [1, 19, 66]. Fe and Pb (both of which may interact with hemoglobin) were separated from the other metals to constitute PC3. The high concentration of Pb results in anemia, inhibits some enzymes, and chelates with Fe [34, 67].

### 3.6. Application of Pb Isotopes in Biological Fractionation

**Figure 3:**
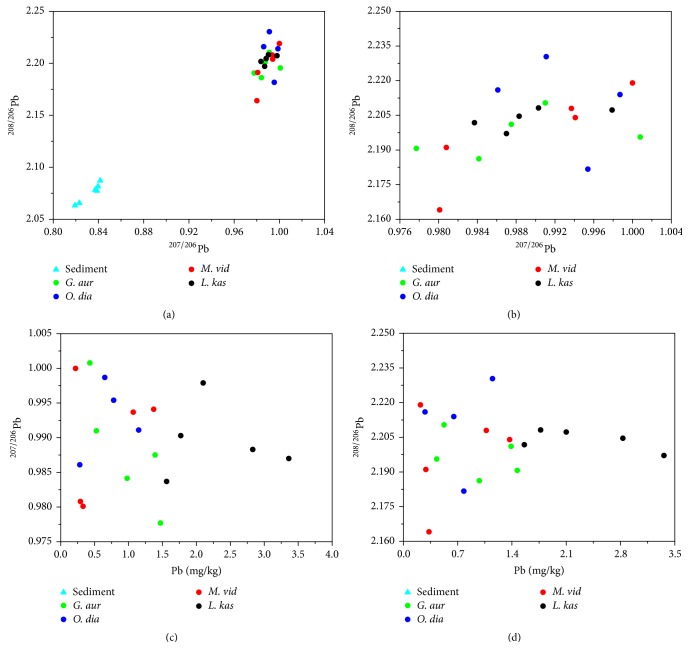
^208/206^Pb and ^207/206^Pb in the four fish species and sediment (Indigo triangle = sediment in South China Sea (from [45]); colored circles = fish species).

The Pb concentrations in most fish species were lower than 2 mg/kg (Figures 3(c) and 3(d)), except* L. kas*. All tissues of* L. kas* showed a homogeneous distribution of ^208/206^Pb with a mean of 2.204, while its ^207/206^Pb varied from 0.984 to 0.998 and the maximum value was detected in the liver. In* G. aur*, ^207/206^Pb decreased with increasing Pb concentration. The gill and liver had low Pb concentrations and ratios, while those in the intestine and muscle were both high. The ovary of* M. vid* which had a low Pb concentration, however, had higher ^207/206^Pb ratios than the other tissues.

There was a large variation of Pb isotopic ratios among different tissues (Figure 3(b)), which was similar to other mammal or birds [32]. The highest concentration of Pb measured in the gill suggested that the fish can accumulate Pb from water primarily through its gill [68]. Compared with the Pb isotopic ratio in their surrounding environments (Figure 3(a)), the studied fish tended to accumulate Pb with higher atom weight, which resulted in higher ratios in different tissues. These results suggested that biological fractionation was present in these fish species.

### 3.7. Human Consumption Risk Assessment

Metals can enter the human body by consumption of the muscle of fishes, and so the metal concentrations in the muscle can be used as a tool to assess human health risks [3]. The Food and Agriculture Organization (FAO) and the World Health Organization (WHO) propose the permissible limit of metals to evaluate human risk from food consumption, and the values are as follows: 100 mg/kg for Fe, 0.5 mg/kg for Mn, 2.0 mg/kg for Pb, 0.15 mg/kg for Cr, 10.0 mg/kg for Cu, 50 mg/kg for Zn, and 0.5 mg/kg for Cd [48, 49]. Cr and Mn concentrations in all fish species in our study exceeded the permissible limit values. The values of Zn in* O. dia* and Fe in* M. vid* were also above the FAO/WHO guidelines.

The results of MSC_*X*_ are listed in Table 7. According to an FAO report, per capita fish consumption in China is 104 g/day [69]. The MSC values of metals in female* G. aur* were higher than those in male* G. aur*, except for Ni. The* O. dia* suffered severe Zn pollution and would pose danger to human health, because the MSC_Zn_ in* O. dia* was below 100 g fish/day. This meant that, in order to avoid the negative effect of Zn, per capita fish consumption of* O. dia* from Yongshu Island should be below 60.33 g/day. Both MSC_Fe_ and MSC_Cu_ in all fish species exceeded 1000, especially for Fe in* O. dia*, which had the highest value of MSC. This meant that Fe and Cu are likely to cause less harm to human health by fish consumption. The lowest values of MSC in* G. aur*,* M. vid*, and* L. kas* were 300 g/day for Cd, 159.02 g/day for Pb, and 121.02 g/day for Pb, all higher than the per capita fish consumption.

According to the results from the assessment, MSC_Zn_ and MSC_Pb_ were lower than the values of other metals MSC, which indicated that Zn and Pb may cause more harm to human by fish consumption. The muscle of* O. dia*,* M. vid*, and* L. kas* tended to accumulate higher concentrations of Zn and Pb. These results indicated that the safe consumption of* O. dia*,* M. vid*, and* L. kas* should be below the MSC, while the concentrations of metals in* G. aur* were safe for consumption.

## 4. Conclusion

This study presented the variations of metals in different tissues of four species of fish. Fe and Zn showed the highest concentrations in all species, while the value of Pb in* L. kas* was generally higher than in the other species (*p* < 0.01). The gender difference significantly impacted on the distribution of metals; for example, the concentrations of metals in female* G. aur* were higher than those in the male. More metals were accumulated in the intestine and liver, compared with other tissues. In addition, dietary exposure was more important than water exposure for food uptake and metal accumulation in fish from Yongshu Island waters.

According to the results of statistical analysis, Zn, Mn, and Ni acted as essential metals that showed a significant positive relationship (*p* < 0.01). Cu and Cd also had a positive correlation and made up PC2, which competed to form stable chelates. Fe and Pb constituted PC3, and both interacted with hemoglobin.

Pb isotopic ratios varied among different tissues in the studied fish, but all showed higher Pb isotopic ratios than those in their surrounding environments, which indicated the biological fractionation present in these species. Bearing in mind the safe fish consumption levels for humans, the MSC_Zn_ in* O. dia* was below 100 g fish/day and the MSC_Pb_ in* M. vid* and* L. kas* were slightly above 100 g fish/day, suggesting that this may cause a health risk to human health since consumption would be above the safe threshold.

## Figures and Tables

**Table 1:** Detection limit of ICP-MS and analytical results of standard reference material (cod, DORM-4).

Metal	Detection limit (ng/L)	Measured value (mg/kg)	Standard value (mg/kg)	Recovery (%)
Cu	3.20	16.6	15.9 ± 0.9	104
Pb	1.20	2.60	2.4 ± 0.3	108
Zn	2.33	56.9	52.2 ± 3.2	109
Cd	1.20	32.3	30.5 ± 1.6	106
Cr	12.0	5.67	5.2 ± 0.7	109
Mn	33.2	256	279 ± 14	92
Ni	3.20	1.55	1.4 ± 0.2	114
Fe	731	363	341 ± 27	106

**Table 2:** Concentrations (mg/kg, dry weight) of eight metals and ^207/206^Pb and ^208/206^Pb in female and male* Gnathodentex aureolineatus (G. aur)*.

Gender	Number	Total length (cm)	Weight (g)	Tissues	Metals (mg/kg)								^207/206^Pb	^208/206^Pb
					Fe	Mn	Zn	Ni	Cu	Pb	Cd	Cr		
Female	10	17.38 ± 0.47	72.64 ± 1.45	Muscle	43.8	1.09	13.3	0.34	0.55	0.65	0.12	1.83	0.991	2.219
				Gill	146	9.27	92.6	0.58	1.49	1.94	0.29	1.51	0.982	2.194
				Liver	804	4.34	555	2.86	22.4	0.82	21.6	1.66	0.979	2.188
				Intestine	157	6.89	104	1.05	6.11	1.18	4.15	1.92	0.987	2.191
				Ovary	105	2.12	328	2.12	10.0	0.43	2.39	2.05	1.001	2.196

Male	6	17.67 ± 0.40	70.72 ± 8.20	Muscle	16.6	1.11	6.04	0.49	0.42	0.40	0.08	1.53	0.991	2.202
				Gill	258	7.98	40.3	0.54	0.98	1.00	ND	1.66	0.974	2.188
				Liver	493	5.81	122	2.98	15.8	1.96	21.55	4.33	0.996	2.214
				Intestine	115	8.60	47.5	1.85	6.30	0.78	5.25	2.16	0.982	2.182

ND: below the detection limit.

**Table 3:** Concentrations (mg/kg, dry weight) of metals and ^207/206^Pb and ^208/206^Pb in muscle, gill, liver, intestine, and ovary in three fish species (*O. dia*, *M. vid,* and *L. kas*).

Fish	Total length (cm)	Tissues		Metals (mg/kg)							^207/206^Pb	^208/206^Pb
			Fe	Mn	Zn	Ni	Cu	Pb	Cd	Cr		
*Oxycheilinus diagrammus* (*O. dia*)	22 ± 2	Muscle	14.9	1.19	99.4	0.78	1.35	0.78	0.06	1.61	0.995	2.182
		Gill	91.5	9.76	55.7	1.20	2.45	1.15	0.35	1.88	0.991	2.230
		Liver	1405	3.40	48.8	0.50	12.8	0.28	8.12	1.72	0.986	2.216
		Intestine	645	3.37	110	0.86	12.2	0.65	10.19	3.80	0.999	2.214

*Melichthys vidua *(*M. vid*)	19.9 ± 1.4	Muscle	109	1.92	13.8	0.35	1.22	1.37	0.14	1.21	0.994	2.204
		Gill	125	10.21	73.3	0.75	1.72	0.33	0.25	3.32	0.980	2.164
		Liver	674	18.27	287	0.50	6.89	0.29	10.2	1.91	0.981	2.191
		Intestine	250	51.83	1296	3.28	11.0	1.07	5.21	3.93	0.994	2.208
		Ovary	119	11.83	399	1.04	3.94	0.22	0.60	4.13	1.000	2.219

*Lutjanus kasmira* (*L. kas*)	19.2 ± 1.2	Muscle	75.9	1.01	17.5	0.30	1.05	1.77	0.03	2.01	0.990	2.208
		Gill	534	8.95	54.6	0.34	1.33	3.36	0.10	2.11	0.987	2.197
		Liver	659	5.34	358	1.12	12.6	2.10	7.81	4.87	0.998	2.207
		Intestine	384	3.89	68.3	1.41	8.82	2.83	4.96	4.60	0.988	2.205
		Ovary	242	2.29	432	0.40	3.98	1.56	0.49	1.60	0.984	2.202

*Note*. (1) The data relating to *G. aur* is set out in Table 2; and (2) the ovaries of female *G. aur* and *O. dia* were too small to obtain data.

**Table 4:** Metal concentrations in fishes from the SCS and other areas and background values (aerosol and sediments) in the SCS (mg/kg).

Sample	Area	Fe	Mn	Cd	Cr	Cu	Ni	Pb	Zn	Reference
4 species	Yongshu Island	14.89–108.91	0.92–1.92	0.03–0.14	1.21–2.00	0.42–1.35	0.34–0.78	0.4–2.27	6.04–99.45	This research
6 species	Northeast Mediterranean Sea	19.60–78.40	—	0.37–0.79	1.56–2.42	2.34–4.41	—	2.98–6.12	16.5–37.4	[8]
4 species	Western SCS			0.006–0.05	0.18–0.85	0.12–0.77	0.11–0.25	0.13–0.68	2.41–4.73	[39]
Fishes	Pearl River Estuary	—	—	0.01–0.13	0.11–4.27	0.15–7.55	0.17–2.08	0.09–30.7	8.78–30.26	[40]
*Lutjanus johnii*	Mumbai Harbor, India	62.31	2.08	0.07	0.47	1.88	—	ND	25.55	[41]
*Geophagus brasiliensis*	South of Brazil	20.37 ± 19.47	6.75 ± 1.60	0.002 ± 0.001	4.02 ± 1.68	27.55 ± 8.65	3.70 ± 1.35	1.91 ± 1.31	23.18 ± 4.48	[42]
*Leuciscus cephalus*	Yenicaga Lake, Turkey	16.03 ± 4.78	2.50 ± 1.38	ND	0.16 ± 0.05	1.79 ± 0.55	0.06 ± 0.01	0.46 ± 0.17	57.81 ± 21.5	[43]
*Tinca tinca*	Yenicaga Lake, Turkey	9.23 ± 4.09	2.55 ± 0.79	0.01 ± 0.02	0.16 ± 0.09	1.42 ± 0.26	0.34 ± 0.11	0.68 ± 0.20	45.53 ± 9.11	[43]
Aerosol^*∗*^	Northern SCS	176 ± 130	0.98 ± 1.54	0.065 ± 0.044	1.53 ± 1.04	6.94 ± 3.27	0.94 ± 0.615	3.70 ± 3.01	13.3 ± 4.98	[5]
Sediments^*∗*^	SCS	—	—	0.4–1.3	6.8–67.4	4–19	7.8–26.1	15.1–36.1	32–74	[44].

ND: below the detection limit; —: no data; ^*∗*^background value.

**Table 5:** Pearson correlation coefficients between metals in different species.

	Fe	Mn	Zn	Ni	Cu	Pb	Cd	Cr
Fe	1	0.023	0.034	0.051	0.141	0.039	0.316	0.164
Mn		1	0.795^*∗∗*^	0.568^*∗∗*^	0	−0.100	0.013	0.309
Zn			1	0.622^*∗∗*^	0.239	−0.150	0.218	0.447^*∗*^
Ni				1	0.091	−0.004	0.211	0.349
Cu					1	−0.211	0.934^*∗∗*^	0.133
Pb						1	−0.188	0.006
Cd							1	0.148
Cr								1

^*∗*^ *p* < 0.05; ^*∗∗*^*p* < 0.01.

**Table 6:** Principal component analysis (PCA) of metals in the four fish tissues.

	Components		
	1 (35.323%)	2 (23.633%)	3 (13.829%)
Fe	0.232	0.318	0.664
Mn	0.718	−0.515	−0.113
Zn	0.861	−0.324	−0.115
Ni	0.726	−0.333	0.058
Cu	0.527	0.782	−0.116
Pb	−0.225	−0.224	0.716
Cd	0.572	0.787	0.025
Cr	0.576	−0.167	0.332

**Table 7:** The maximum safe consumption of metals (MSC_A_) in the four fish species (g fish/day).

	Fe	Mn	Zn	Ni	Cu	Pb	Cd
*G. aur* (F)	6575.34	440.38	450.45	882.35	5454.55	329.54	250.00
*G. aur* (M)	17318.10	432.43	993.38	612.24	7142.86	535.50	375.00
*G. aur* (A)	9530.11	436.36	619.83	714.29	6122.45	404.15	300.00
*O. dia*	19341.84	403.36	60.33	384.62	2222.22	274.62	500.00
*M. vid*	2644.39	250.00	433.53	857.14	2459.02	159.02	214.29
*L. kas*	3795.97	475.25	343.45	1000.00	2857.14	121.02	1000.00

F: female; M: male; A: average.
